# Grief, Mindfulness and Neural Predictors of Improvement in Family Dementia Caregivers

**DOI:** 10.3389/fnhum.2019.00155

**Published:** 2019-05-14

**Authors:** Felipe A. Jain, Colm G. Connolly, Leonardo C. Moore, Andrew F. Leuchter, Michelle Abrams, Ramzi W. Ben-Yelles, Sarah E. Chang, Liliana A. Ramirez Gomez, Nora Huey, Helen Lavretsky, Marco Iacoboni

**Affiliations:** ^1^Depression Clinical and Research Program, Department of Psychiatry, Massachusetts General Hospital, Harvard Medical School, Boston, MA, United States; ^2^Department of Biomedical Sciences, College of Medicine, Florida State University, Tallahassee, FL, United States; ^3^Department of Psychiatry and Biobehavioral Sciences, UCLA Semel Institute for Neuroscience and Human Behavior, David Geffen School of Medicine, University of California, Los Angeles, Los Angeles, CA, United States; ^4^Institute for Simulation and Training, University of Central Florida, Orlando, FL, United States; ^5^Department of Psychiatry and Behavioral Sciences, Stanford University, Palo Alto, CA, United States; ^6^Department of Neurology, Massachusetts General Hospital, Harvard Medical School, Boston, MA, United States; ^7^Department of Psychiatry, University of California, San Francisco, San Francisco, CA, United States; ^8^Ahmanson-Lovelace Brain Mapping Center, Brain Research Institute, University of California, Los Angeles, Los Angeles, CA, United States

**Keywords:** grief, dementia, caregivers, mindfulness, depression, functional magnetic resonance imaging

## Abstract

**Background**: Family dementia caregivers often suffer from an immense toll of grief while caring for their loved ones. We sought to identify the clinical relationship between grief, depression and mindfulness and identify neural predictors of symptomatology and improvement.

**Methods**: Twenty three family dementia caregivers were assessed at baseline for grief, mindfulness and depression, of which 17 underwent functional magnetic resonance imaging (fMRI). During fMRI, caregivers were shown faces of either their dementia-stricken relative or that of a stranger, paired with grief-related or neutral words. In nine subjects, post fMRI scans were also obtained after 4 weeks of either guided imagery or relaxation. Robust regression was used to predict changes in symptoms with longitudinal brain activation (BA) changes as the dependent variable.

**Results**: Grief and depression symptoms were correlated (*r* = 0.50, *p* = 0.01), and both were negatively correlated with mindfulness (*r* = −0.70, *p* = 0.0002; *r* = −0.52, *p* = 0.01). Relative to viewing strangers, caregivers showed pictures of their loved ones (picture factor) exhibited increased activation in the dorsal anterior cingulate gyrus and precuneus. Improvement in grief but not mindfulness or depression was predicted by increased relative BA in the precuneus and anterior cingulate (different subregions from baseline). Viewing grief-related vs. neutral words elicited activity in the medial prefrontal cortex and precuneus.

**Conclusions**: Caregiver grief, depression and mindfulness are interrelated but have at least partially nonoverlapping neural mechanisms. Picture and word stimuli related to caregiver grief evoked brain activity in regions previously identified with bereavement grief. These activation foci might be useful as biomarkers of treatment response.

## Introduction

Grief, broadly characterized as the psychological reaction to the perception of loss, is a normal emotional response unique to each individual and circumstance that can include characteristics such as shock and disbelief, sadness and low mood, frustration and anger, anxiety and fear and longing for the lost object (Bowlby, 1973; Rando et al., 2012). Much research has focused on grief secondary to bereavement (the death of a close attachment), particularly when such grief leads to intense yearning and loneliness (complicated grief) or is associated with an episode of Major Depressive Disorder (Prigerson et al., 1995; Bandini, 2015). Serious grief reactions may also occur prior to death when observing and caring for a loved one afflicted with a serious illness such as dementia, and this type of grief has been termed “predeath grief” (Collins et al., 1993; Lindauer and Harvath, 2014). In family dementia caregivers, predeath grief reactions can become prolonged and serially compounded as dementia progresses and losses accrue (Meuser and Marwit, 2001; Chan et al., 2013; Blandin and Pepin, 2017).

Pearlin et al. (1990) developed one of the most widely used heuristics for understanding how losses contribute to negative health outcomes in caregivers, the “Stress Process Model.” The losses caregivers experience are endemic to primary and secondary stressors of the model. Primary stressors include loss of the relationship with the care recipient (CR) due to cognitive decline and behavioral changes; secondary stressors include losses related to role conflicts with work and other activities, as well as a loss of sense of self due to the consuming nature of caregiving. Measures of predeath caregiver grief that account for these losses strongly correlate with caregiver burden (Liew et al., 2018), even when accounting for behavioral problems in the dementia patient and caregiver depression (Holley and Mast, 2009). High levels of caregiver grief are associated with depression and caregiver strain (Chan et al., 2017), and with negative health outcomes post death of the CR (Givens et al., 2011; Shuter et al., 2014).

Several authors have emphasized that grief and its resolution involve many stages (Kübler-Ross, 1969; Blandin and Pepin, 2017). Common to these models is a need to recognize and accept that the loss has occurred, tolerate the emotions associated with it, and adjust or adapt to the loss. Psychological characteristics that have been studied in association with recovery from grief include resilience (Ong et al., 2006) and emotional stability (Mancini et al., 2015), whereas maladaptive dependency traits such as attachment avoidance and attachment anxiety are associated with prolonged grief (Denckla et al., 2011; Mancini et al., 2015).

“Mindfulness” is a multidimensional construct originally drawn from Buddhism (Anālayo, 2003) and simplified for clinical use with patients suffering from stress related disorders by Kabat-Zinn (1994), who defined it conceptually as “paying attention in a particular way: on purpose, in the present moment, and non-judgmentally.” Substantial evidence suggests that mindfulness therapies, which incorporate meditative practice, yoga and mindfulness philosophy, are beneficial for depression and anxiety symptoms in general (Hofmann et al., 2010) and clinical populations (Jain et al., 2015; Kuyken et al., 2016; Goldberg et al., 2018). Although there is no research consensus definition of mindfulness (Van Dam et al., 2018), one of the most frequently used mindfulness inventories (the Five Factor Mindfulness Questionnaire) identifies five components including acting with awareness, observing and describing present-moment sensations and feelings, and responding non-reactively and non-judgmentally to internal and external experience (Baer et al., 2008). Theoretically, such mindfulness qualities should help with coping with negative perceptions related to the serial and compound losses of caring and result in overall lower levels of caregiver grief.

Interest in the application of mindfulness therapies for family caregivers has recently grown. Several studies and systematic meta-analyses suggest that such therapies may be beneficial for reducing measures of caregiver depression and burden (Franco et al., 2010; Whitebird et al., 2013; Norouzi et al., 2014; Brown et al., 2016; Liu et al., 2017; Collins and Kishita, 2018), although not all studies have found these benefits (Oken et al., 2010) and the overall evidence base is small. Our group has presented preliminary evidence from open-label trials that family dementia caregivers may reduce negative affect and increase positive affect and relationship functioning with guided imagery and mindfulness techniques that specifically focus on mentalizing relational content (Jain et al., 2014; Jain and Fonagy, 2018; Sikder et al., 2019). The present study of family caregiver grief was conducted as an add on to the first controlled pilot feasibility study of Mentalizing Imagery Therapy (MIT), the primary goal of which was to determine an effect size of MIT relative to a control condition consisting of progressive muscle relaxation (PMR) for caregiver depression (Jain et al., manuscript in preparation).

We undertook the present investigation to determine the relationship between grief, depression and mindfulness, and to identify their neural underpinnings in the caregiver population. We hypothesized that caregiver predeath grief and mindfulness would be negatively correlated. Because caregivers often perceive the changes in their loved ones as losses of core elements of the CRs’ self or personhood, we also hypothesized that the neural response to grief-related stimuli in caregivers at baseline would overlap with that previously identified cross-sectionally in bereaved individuals, specifically, cortical midline structures including the anterior cingulate and precuneus (Gündel et al., 2003).

## Materials and Methods

### Participants

Participants from a pilot feasibility trial of guided imagery and mindfulness (MIT) vs. relaxation for dementia caregivers (ClinicalTrials.gov/ #NCT02122068) were invited to participate in functional magnetic resonance imaging (fMRI) if they had no contraindications. All procedures were approved by the Institutional Review Board of the University of California, Los Angeles #13-001877, and written informed consent was obtained. Participants were recruited with flyers, newsletter advertisements and presentations at local support groups. Inclusion criteria were being the primary source of assistance or support for the CR, Patient Health Questionnaire Score >9 (indicating elevated depressive symptoms; Kroenke et al., 2001), in contact with the CR at least three times per week for no less than 1 year, a relative of the CR, and English language fluency. Exclusion criteria were active suicidal plan or suicide attempt within the past month, excessive use of alcohol, ideas of harm toward the CR, current violence, Adult Protective Services report on file, diagnosis of schizophrenia or any psychotic disorder, mania, alcohol or drug dependance, any pervasive developmental disorder or cognitive disorder (according to DSM-IV) criteria, being diagnosed as medically unstable, delirious, or terminally ill; a history of skull fracture or cranial surgery entering the calvarium, space occupying intracranial lesion, stroke, aneurysm, Parkinson’s disease, Huntington’s disease, or multiple sclerosis. Participants could not be practicing meditation, guided imagery, or yoga, more than once per week. Twenty-three of the 26 subjects in the trial completed self-report questionnaires for grief and mindfulness and were included in the clinical correlations.

Subjects were assigned by convenience to receive 4 weeks of MIT (Jain and Fonagy, 2018), in which they participated in weekly group meetings and practiced take-home exercises, or a relaxation group to simulate a minimal clinical intervention consisting of provision of a compact disk with a PMR recording. Due to funding limitations and contraindications to magnetic resonance imaging (MRI) in some subjects who provided clinical data, MRI data from the grief task were obtained on 19 subjects at baseline and from a subset of 10 of these subjects at follow up (seven who received guided imagery and three who received a relaxation CD). Of these, two subjects at baseline and one subject at follow up were excluded due to excessive motion artifact, leaving 17 subjects analyzable at baseline and nine subjects at follow up. Because a limited number of subjects were able to receive task-related studies of the neural underpinnings of grief, fMRI data from both groups were combined in the analysis at baseline prior to group assignment, and at 4-week follow up. Combining the groups enabled us to identify candidate neural mechanisms of grief and its resolution, not specific group effects related to MIT or PMR. As this was, to our knowledge, the first fMRI study in a population of caregivers, and longitudinal neuroimaging data is difficult to obtain in this population due to caregiver time constraints for participating in a study and contraindications to MRI in an aging cohort, our rationale was to include as much data as possible to identify overall effects related to clinical change.

### Measures

#### Diagnostic Status

The presence of major depression (and other axis I disorders) was established according to DSM-IV criteria with the MINI International Neuropsychiatric Interview (Sheehan et al., 1998).

#### Self-report Questionnaires

Self-reported symptoms were measured as follows: grief with the Marwit and Meuser Caregiver Grief Inventory—Short Form (Marwit and Meuser, 2005); depression with the Quick Inventory of Depression Symptoms—Self Report (QIDS; Rush et al., 2003); and trait mindfulness with the Five Factor Mindfulness Questionnaire (Baer et al., 2008).

#### fMRI Task

**Figure 1:**
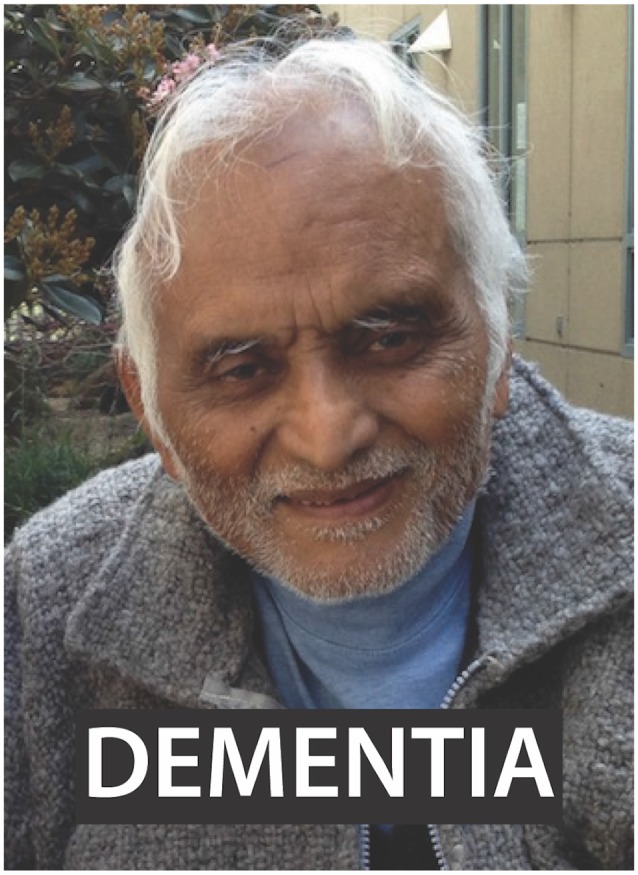
Picture-word composite stimulus example used for task. Subjects were presented with images of either their relative or a stranger with a word with grief connotation or neutral connotation overlaid in a 2 × 2 design. Written informed consent for the publication of the image was obtained.

In the MR system, caregivers were shown picture-word composites of their loved one or a stranger matched for age, sex, and race, together with grief related or emotionally neutral words, in a 2 × 2 design (Figure 1), as previously described (Gündel et al., 2003). Each caregiver provided a photograph of his or her CR suffering from dementia, and these photographs were digitized and cropped to focus only on the face. To identify sex, race and approximate age-matched photographs of strangers, a member of the study staff searched online and selected an image that was confirmed by the caregiver to be a stranger whom they had not previously seen. The stranger images were cropped to make the stranger and relative faces of comparable size. Fifteen words related to grieving (such as disease, dementia, and sick) were chosen and matched for part of speech and length to 15 emotionally neutral words (such as village, planter and curve). Composites were pseudo-randomly generated such that every word appeared once with every picture (60 total stimuli) for a given presentation. After 10 s of fixation on a cross, picture-word composites appeared for 5 s, randomly interleaved with 26 inter-trial intervals during which the fixation cross appeared for 5 s (Total task time 440 s). Caregivers were instructed to view the composites and observe any emotional reactions they might have. After the scan, subjects completed a Likert scale from 1 to 5 (1 = “Not at All”; 3 = “Moderately”; 5 = “Very Much”) to assess how well the words were related to their experience of caregiver grief.

#### MR Data Acquisition

Echo-planar images (EPIs) T2* images were acquired on a 3T Siemens Trio Tim (Erlangen, Germany) MRI system at the UCLA Ahmanson-Lovelace Brain Mapping Center or the UCLA Staglin IHMRO Center for Cognitive Neuroscience. For each subject, pre and post scans were always obtained on the same scanner. Data acquisition consisted of one functional EPI scan: Time of Echo (TE)/Time of Relaxation (TR) = 28 ms/2,000 ms, flip angle = 90°, Field of View (FOV) = 192 mm, 64 × 64 matrix, 3 × 3 mm in-plane resolution, 34 4 mm axial slices. A high-resolution structural T1-weighted MPRAGE scan (TE/TR = 4.9 ms/TR = 11.6 ms, flip angle = 8°, FOV = 256 mm, slice thickness 1 mm, matrix 256 × 256 × 180) was acquired to permit functional localization and normalization to standard space.

### Statistical Analysis

#### Questionnaires

In cases of missing responses to single items on a questionnaire (<3% of all responses), interpolation was performed with the average score of the rest of the items on that questionnaire. Scales were discarded if more than two questions had not been answered. Questionnaire data were examined for normality by examination of Quantile-Quantile plots and using the Shapiro-Wilk test, and both visual inspections of the plots and negative Shapiro-Wilk test (*p* > 0.10 for all questionnaires) indicated the data conformed to normality. Total scores for each questionnaire were computed and Pearson correlations were performed among the self-report scales as well as between self-report scales and number of caregiving years and hours per week. Differences in grief relevance between grief and neutral words were assessed with unpaired Wilcoxon signed-rank test. Linear regression was used to determine whether the ratings of grief words predicted grief symptoms, mindfulness and depression. Levels of symptoms in the nine subjects with neuroimaging follow-up data were provided to illustrate the magnitude of clinical symptom change correlating with neuroimaging results: effect size, median change score and range of change scores were presented. Effect size (*d*) was computed as mean of the change scores divided by standard deviation of the change scores. Behavioral data averages were presented as mean ± standard deviation. Analyses were conducted in R version 3.3.2 (R Core Team, 2016). Figures were created using R, Analysis of Functional Neuroimages (AFNI; Cox, 1996) and Adobe Illustrator (Adobe Inc, San Jose, CA, USA).

#### fMRI Analysis

All image analysis was conducted using AFNI. T1-weighted images were skull-stripped and transformed to MNI152 standard space using both affine and non-linear transforms. EPI data were despiked, time-shifted, motion corrected and aligned to standard space (MNI152 at 3 × 3 × 3 mm) in a single operation to minimize added smoothing (Saad et al., 2009). Data were subsequently blurred so that the effective smoothness was 8 mm FWHM using 3dBlurToFWHM and then scaled to have a mean of 100. Censoring of volumes where the Euclidean norm of the six motion estimates was greater than 0.3 was performed. While we adopted a rule that subjects with more than 30% of their time-series censored would be excluded from further analysis, no subjects crossed this threshold and therefore none were excluded on this basis. Outlier volumes where more than 20% of voxels were greater than the median absolute deviation of the detrended time-series were also censored. The average number of censored volumes was 10 ± 15 (range 0–52). Preprocessed time-series were subjected to generalized least squares regression that estimates the serial correlation structure of the noise with an ARMA(1, 1) model. Four time-series of interest were defined, one for each picture-word composite type: relative-grief, relative-neutral, stranger-grief, and stranger-neutral. These were convolved with a prototypical hemodynamic function prior to regression. Two general linear tests (GLTs) were computed within participant; relative vs. stranger, and grief vs. neutral. All time points not accounted for by regressors constituted the baseline. Also included in the baseline were six motion estimates and their temporal derivatives in addition to a 3rd order Legendre polynomial to model slow signal drift.

To relate differences in processing stimuli type to clinical measures, voxel-wise robust linear regression (Venables and Ripley, 2002) was performed in R. Robust regression is inherently more conservative and resistant to the presence of outliers in data (Huber, 1964) and as such has been recommended for use in neuroimaging data (Wager et al., 2005). Robust regression to predict baseline symptoms was performed by calculating the difference in brain activation (BA) for each contrast and using this as the independent variable, with the rating scale total score as the dependent variable:

$${\text{Scale}}_{\text{Total}}={\text{m(BA}}_{\text{contrast 2}}-{\text{BA}}_{\text{contrast 1}}\text{)}+\text{b}$$

For the picture factor, contrast 2 was the picture of the relative and contrast 1 was picture of the stranger; whereas for the word factor, contrast 2 was the grief word and contrast 1 the neutral word. To predict changes in symptoms, the formula was adopted as follows:

$$({\text{Scale}}_{\text{Total }Post}-{\text{Scale}}_{\text{Total }Pre})=\text{m}\left[(\text{B}{\text{A}}_{\text{contrast 2 }Post}\right]-{\text{BA}}_{\text{contrast 1 }Post})-(\text{B}{\text{A}}_{\text{contrast 2 }Pre}-\text{B}{\text{A}}_{\text{contrast 1 }Pre})]+\text{b}$$

Thus, if BA differentiation between contrast 2 and contrast 1 increased after the intervention, the independent term representing neural activity change would increase (regardless of the absolute change of BA).

#### Thresholding and Correction for Multiple Comparisons

Task-based analysis and robust regressions were each required to pass voxel-wise two-tailed statistical thresholds (voxel-wise *p* < 0.01; task analysis *z* = 2.58, baseline regression *t*_(13)_ = 3.01, follow-up regression *t*_(7)_ = 3.50). Voxels were further required to be part of a cluster of minimum volume, determined by a Monte-Carlo method, to control for multiple comparisons. For the task analysis, the smoothing was estimated separately for each *t*-test by using a permutation test. In combination, these methods have been shown to accurately control the false positive rate (Cox et al., 2017). For the regression analyses, the smoothing of the data was accounted for using the average of the spatial auto-correlation function (ACF) parameters from the individual subject’s data (Cox et al., 2017). Alpha was set at *p* < 0.05 and was further Bonferroni corrected for the number of comparisons. For the task-analysis, the minimum cluster sizes were 2,106 μL for relative vs. stranger and 2,295 μL for grief vs. neutral at a corrected *p* < 0.025 (= 0.05/2). For the regression analyses, clusters were Bonferroni corrected for six comparisons: three questionnaires × two factors, *p* < 0.0083, minimum cluster size of 1,269 μL for baseline regression and 1,242 μL for follow-up regression analysis.

## Results

### Sample (Table 1)

The subjects were largely elderly, female and college educated. Approximately 25% of participants were minority. They had on average been performing caregiving responsibilities for 6 years and for many, informal caregiving responsibilities approached those of a full-time job. The overall sample and neuroimaging subsamples were demographically comparable. For the conjoint follow-up imaging analysis (Sample 3, Table 1), data from two subjects were available from the relaxation group and seven from the guided imagery group. The two subjects were 55 and 79 years old, both female, and with baseline QIDS scores of 8 and 13; while those in guided imagery (*n* = 7) ranged in age from 38 to 77 (mean age 60), were 86% female and had baseline QIDS from 10 to 31 (mean 19).

**Table 1:** Baseline demographic and clinical variables.

	Sample 1 (*N* = 23)	Sample 2 (*N* = 17)	Sample 3 (*N* = 9)
Age	60 ± 11	60 ± 11	60 ± 13
% Female	91	88	82
Ethnicity			
% White	74	65	56
% Hispanic	13	18	22
% African American	13	18	22
Education (years)	16 ± 2	16 ± 2	15 ± 2
Years Caregiving	6 ± 4	6 ± 4	7 ± 4
Caregiving Hours Per Week	28 ± 21	25 ± 24	29 ± 28
Live with Recipient (%)	65	53	55
Dementia Relative			
Spouse (%)	30	35	27
Parent (%)	70	65	73
Major Depression (%)	83	83	82
Generalized Anxiety Disorder (%)	35	47	55
Grief	58.1 ± 11.7	59.0 ± 12.9	61.7 ± 13.8
QIDS—Depression	14.2 ± 7.1	14.0 ± 8.3	17.2 ± 8.1
Mindfulness	128.3 ± 24.3	124.7 ± 20.8	122.3 ± 19.9

*Sample 1 consisted of participants with symptom questionnaires at baseline. Sample 2 was a subset of Sample 1 with baseline task grief neuroimaging. Sample 3 was a subset of Sample 2 that received neuroimaging at baseline and 4 weeks follow-up*.

### Baseline Symptom Correlations

**Figure 2:**
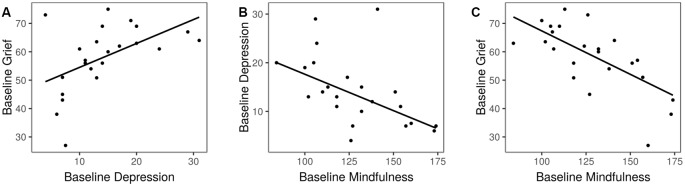
Associations of baseline psychological variables. **(A)** Depression and grief. **(B)** Mindfulness and depression. **(C)** Mindfulness and grief.

At baseline, subjects exhibited levels of grief comparable to those seen on average in dementia caregivers (score of 58.1 ± 11.7; Marwit and Meuser, 2005). Eighty percentage of subjects were diagnosed with a major depressive episode, and depression symptoms on the QIDS were on average of moderate severity (please refer to Sample 1 in Table 1). Grief was correlated with depression (*r* = 0.50, 95% CI 0.11–0.76, *p* = 0.01, Figure 2A). Mindfulness exhibited a moderate negative correlation with depression (*r* = −0.52, 97% CI −0.77 to −0.14, *p* = 0.01, Figure 2B), and a strong negative correlation with grief (*r* = −0.70, 95% CI −0.87 to −0.41, *p* = 0.0002, Figure 2C). There was no relationship between caregiving hours per week or number of years caregiving, and grief or depression symptoms (*p* > 0.4 for all comparisons).

### Relevance of Grief Word Ratings

On a Likert scale, grief words (presented in picture-word composites during the fMRI task, and rated after fMRI) were rated as more highly relevant to the caregivers’ experience of grief than neutral words (3.72 ± 0.35 vs. 1.54 ± 0.37, *p* < 10^−5^). Higher Likert ratings were also associated with higher total grief symptoms (*F*_(1,14)_ = 10.4, *p* = 0.006) and lower mindfulness (*F*_(1,14)_ = 8.2, *p* = 0.01), but were not related to depression (*F*_(1,15)_ = 1.6, *p* = 0.2).

### Symptom Changes

In the nine subjects in which neuroimaging was obtained (Sample 3 in Table 1), grief scores were 61.7 ± 13.8 at baseline and 55.1 ± 15.2 at follow-up (*d* = 0.92, median change = −4.5 points, range = 2 to −19 points). Depression scores were 17.2 ± 8.1 at baseline and 8.5 ± 5.0 at follow-up (*d* = 1.08, median change = −7 points, range 0 to −28 points). Mindfulness scores were 122.3 ± 19.9 at baseline and 129.0 ± 23.6 at follow-up (*d* = 0.51, range −12 to +27 points).

### Brain Response to Stimuli at Baseline (Table 2)

**Figure 3:**
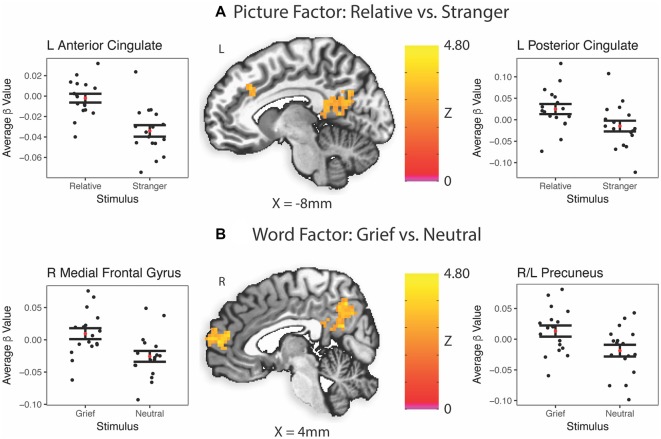
Task-related brain activation (BA). Red dot indicates the mean; error bars are standard error of the mean. ß, Average beta value for each subject across every stimulus presentation of that type; L, left; R, right. **(A)** Picture factor (relative vs. stranger). **(B)** Word factor (grief vs. neutral).

In caregivers undergoing MRI at baseline, the picture factor evoked two clusters of activation that survived multiple comparisons, the first in the left posterior cingulate and precuneus regions, and the second in bilateral anterior cingulate gyri (Figure 3A). The word factor evoked a cluster of activity in bilateral dorsal anterior cingulate cortex and posterior cingulate (Figure 3B) as well as a region of the supramarginal gyrus stretching into the inferior parietal lobe.

**Table 2:** Task-related neuroimaging findings.

				Center of Mass			
Structure	BA	Hemisphere	Volume (mm^3^)	*x*	*y*	*z*	*Z* score
*A. Picture Factor*							
Posterior Cingulate/Precuneus	7, 31	L	2,889	−7	−58	20	3
Anterior Cingulate	31, 32, 24	Bilateral	2,457	−1	29	22	3
*B. Word Factor*							
Precuneus	7	Bilateral	6,021	−1	−58	34	3.1
Medial Frontal Gyrus	10	R	2,538	4	58	11	3.1
Supramarginal Gyrus/Inferior Parietal	40	L	2,079	−52	−50	36	3

*Activation for picture factor (Relative—Stranger contrast) and word factor (Grief words—Neutral Words). BA, Brodmann Area; L, left; mm, millimeter; R, right*.

### Neural Predictors of Symptoms at Baseline (Table 3)

**Figure 4:**
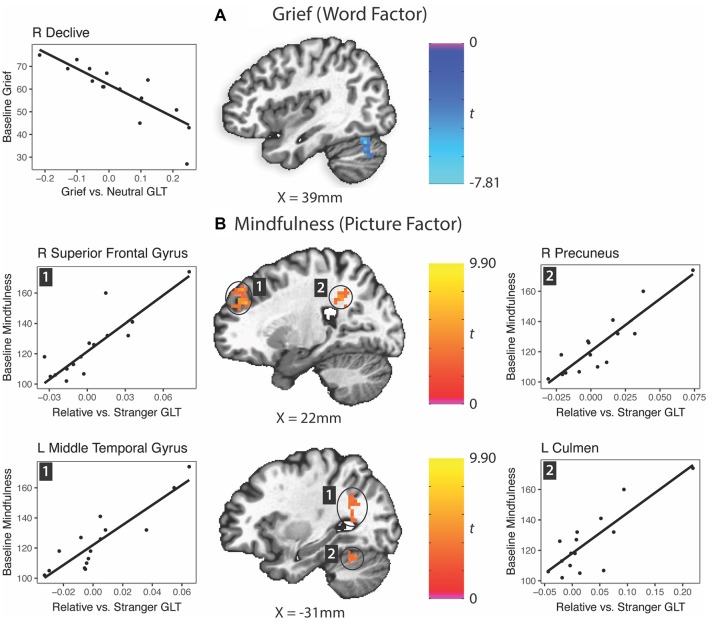
Prediction of grief and mindfulness scores based on word factor (grief word—neutral word) or picture factor (relative—stranger) brain activity responses. GLT, general linear test; L, left; R, right. **(A)** Grief (word factor). **(B)** Mindfulness (picture factor).

A reduction in activation of the right cerebellar declive to grief words vs. neutral words predicted grief at baseline (Figure 4A). Multiple regions responding with greater activity to the picture factor predicted higher mindfulness: a region of the right anterolateral prefrontal cortex, middle temporal gyrus extending into the angular gyrus, right precuneus and left cerebellar culmen (Figure 4B). Regarding depression, no clusters survived Bonferroni correction for multiple comparisons.

**Table 3:** Neural predictors of baseline symptoms.

					Center of Mass				
Factor	Structure	BA	Hemisphere	Volume (mm^3^)	*x*	*y*	*z*	Average *t*	Cohen’s *d*
*A. Grief*									
Word	Right Declive		R	1,296	43	−69	−29	−3.91	−2.2
*B. Mindfulness*									
Picture	Precuneus/Inferior Parietal Lobule	31	R	2,835	27	−46	30	3.86	2.1
Picture	Superior Frontal Gyrus	9	R	1,647	24	42	33	4.13	2.3
Picture	Middle Temporal Gyrus/Angular Gyrus	19, 39	L	1,431	−32	−55	22	3.79	2.1
Picture	Culmen		L	1,377	−28	−55	−23	3.84	2.1

*Brain activation predicting baseline grief and mindfulness to picture factor (Relative—Stranger contrast) and word factor (Grief words—Neutral Words). BA, Brodmann Area; L; left; mm, millimeter; R, right*.

### Changes in Neural Activity Predicting Change in Symptoms (Table 4)

**Figure 5:**
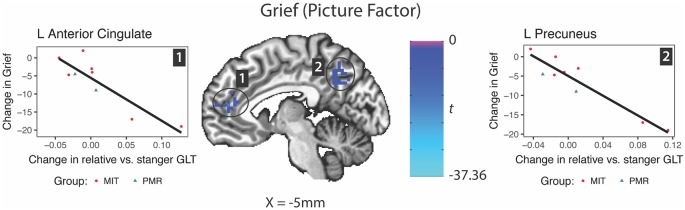
Changes in brain activity evoked by the picture factor (Relative—Stranger contrast) predicting change in grief symptoms. GLT, general linear test; L, left; R, right.

In response to the picture factor, relatively increased activity at follow up in the precuneus and anterior cingulate gyrus predicted improvement in grief (Figure 5). For mindfulness and depression, no clusters reached reporting criteria.

**Table 4:** Neural predictors (picture factor) of change in grief symptoms.

				Center of Mass				
Structure	BA	Hemisphere	Volume (mm^3^)	*x*	*y*	*z*	Average* t*	Cohen’s *d*
Precuneus	7	L	2,268	−6	−61	37	−4.78	−3.6
Anterior Cingulate	32	Bilateral	2,241	−2	44	13	−5.65	−4.3

*Brain activation change in response to picture factor (Relative—Stranger contrast) predicting change in grief symptoms. BA, Brodmann Area; L, left; mm, millimeter; R, right*.

## Discussion

These findings contribute to the literature on the relationship between mindfulness and grief, and extend previous findings on the neural underpinnings of grief as experienced in the setting of bereavement to caregivers experiencing grief before the actual death of their loved one. At least since Bowlby (1973), grief has been recognized evolutionarily as an unresolved attachment, that is, the important object is in some way gone or lost, but the need for that attachment remains within the brain. Within Buddhism, mindfulness represents the ability to calmly observe all experience as fundamentally transitory, and thus to remain unattached and centered in the face of changing mental events (Anālayo, 2003). Conceptualizations of mindfulness within clinical psychology emphasize primacy of capacities to observe non-judgmentally without reactivity in the face of internal mental events (such as feelings and perceptions of loss; Kabat-Zinn, 1994; Baer et al., 2008). The psychological conceptualizations of grief and mindfulness thus have opposing features, and this inverse relationship was also demonstrated by our self-report findings that mindfulness and grief were strongly negatively correlated (*r* = −0.70). We are aware of one other report that quantitatively compared grief and mindfulness, but in a bereaved sample (Huang et al., 2019). This study also found a negative correlation but of lower magnitude (*r* = −0.52) than that observed in this study. We speculate that predeath grief could be more highly related to mindfulness than bereavement-related grief because predeath grief involves a set of continuous stressors that require daily mindfulness to combat, whereas bereavement-related grief is related to a past loss. Post-bereavement, supportive family and societal mourning processes might bolster the mood of grieving individuals (Walsh and McGoldrick, 1998; Neimeyer et al., 2014) and thus diminish the association of mindfulness with grief.

In this study, picture and word stimuli resulting in midline BA that predicted grief symptoms or their improvement did not similarly result in BA that predicted depression or its improvement, suggesting a closer coupling of images of the CR and words related to the dementia caregiving experience with grief than depression. This may be due to depression symptoms resulting from a multifactorial origin (Blazer, 2003), vs. the more specific nature of the grief experience as tied to the relationship with the loved one and losses entailed by it. Our task-based findings of BA to grief stimuli in dorsal anterior cingulate cortex, ventromedial prefrontal cortex, and precuneus are strikingly similar to those previously reported using a similar experimental paradigm that elicited a transient experience of grief in recently bereaved individuals (Gündel et al., 2003; O’Connor et al., 2008).

Activation of the dorsal anterior cingulate cortex in the picture factor comparison might have been due to triggering of a social pain reaction that has been strongly associated with dorsal anterior cingulate function (Eisenberger et al., 2003; Lieberman and Eisenberger, 2015) and which was previously demonstrated to occur with presentation of similar stimuli to bereaved patients (Gündel et al., 2003). Precuneus activation in this picture contrast is likely related to familiarity of the relative vs. stranger image (Gobbini et al., 2004; Ramon and Gobbini, 2018). In the word factor comparison, activation of major nodes of the default mode network, the precuneus and ventromedial prefrontal cortex (Kim, 2012), might be related to evocation of losses related to the loved one’s illness. These word factor regions do not overlap with the neural response to words related to illness or body symptoms in healthy subjects (Mier et al., 2017) or those with health anxiety symptoms (Witthöft et al., 2013), suggesting that the cortical midline activation we observed in caregivers is not related to their own anxiety about dementia.

Despite the strong negative correlation between mindfulness and grief, the neural activations associated with them differed. When viewing grief vs. neutral words, lower grief symptoms were predicted by higher activation of the right declive, close to a region of the right posterior cerebellum identified as involved with grief previously (Gündel et al., 2003). Although the reason for this cerebellar activation is unclear, clinical, lesion and neuroimaging studies have converged on a role for the cerebellum in cognitive and affective control (Sokolov et al., 2017; Hoche et al., 2018). The region predicting grief in our study overlaps with center of mass coordinates previously associated with social but not self-referential processing (Schilbach et al., 2006), compassion (maintaining a positive and benevolent concern for others when viewing suffering; Engen and Singer, 2015), theory of mind and empathy (Völlm et al., 2006). This might indicate that those subjects in which grief words triggered an allocentric neural response were experiencing less grief.

Mindfulness was not predicted by response to grief vs. neutral words in the abstract, but with BA while viewing the relative vs. the stranger images, thus directly relating to the degree of interpersonal familiarity and possibly social attachment. While our clinical data do not allow for concluding a direction of effect, i.e., whether higher levels of mindfulness reduce grief or lower levels of grief allow for a greater mindful experience, or even for firmly concluding that a mechanistic relationship exists, we suggest that the frontal, parietal and cerebellar neural activation patterns are most consistent with a role for mindfulness in regulating the experience of grief. Regions associated with mindfulness included a part of the lateral prefrontal cortex previously associated with mindfulness of breathing (Dickenson et al., 2013), and attention (Corbetta et al., 1998). Both this and the inferior parietal lobule/precuneus region have been associated with working memory (Postle et al., 2004; Brehmer et al., 2011), which may be important for following the task directions to pay attention to the stimuli and emotions that might arise, in the face of strong emotions being triggered. This hypothesis should be confirmed with future experiments that dissect caregivers’ experiential responses to the picture word-composites. Finally, activation of the culmen has previously been associated with the regulation of negative emotions (Belden et al., 2014; Greening et al., 2014).

Somewhat counterintuitively, longitudinal increases in BA to the relative’s image vs. that of the stranger within the precuneus and anterior cingulate predicted improvements in grief. This suggests that the process of recovery from a multifaceted symptom such as grief is not merely due to inactivation of cortical midline structures or a simple “on/off” mechanism but a more dynamic change in the processing of the stimuli that might reflect a remapping of self-referential associations. Indeed, the particular subregion of the precuneus identified here only partially overlapped with that activated by the task at baseline and its center of mass was more superior. In contrast, a longitudinal study in participants receiving Mindfulness Based Cognitive Therapy combined with grief psychoeducation for bereavement associated grief found that greater deactivation of the precuneus during a numeric Stroop task was associated with improvement (Huang et al., 2019). Differences in the direction of precuneus involvement could be due to the effects of treatment, participants, or task conditions. Related to task conditions, the numeric Stroop task taxes executive function, whereas the grief stimuli in the present work would be thought to activate emotional representations and thus differentially modulate the precuneus. Taken together, the findings reinforce the importance of cortical midline structures in grief processing and suggest possible targets for future research.

Strengths of this report inform its limitations. The sample is unique in the literature; to our knowledge, there have not previously been functional imaging studies of caregivers and this population is difficult to study due to time constraints and contraindications to fMRI scanning (such as implants) that are more common in a largely older adult population. To our knowledge, there have also not been longitudinal neuroimaging studies of predeath grief. Another strength was a large range of behavioral scores at baseline and follow up for correlation with neuroimaging data. Limitations of our work include the small sample size at baseline (17 subjects) and follow up (nine subjects), which resulted in low power and precluded inclusion of covariates such as age. Particularly because of the limitations of our pilot funding, we were unable to independently study the mechanism of grief reduction in the two different groups, but rather aggregated the data to determine common brain changes that are associated with improvement. Although this decreases specificity for understanding the particular therapies, it also may increase the generalizability of our findings. Our paradigm did not elicit specific activation related to depression or its improvement, which might be due to less relevance of our stimuli for depression in combination with low statistical power. Because we did not have a control group of non-caregivers experiencing grief, it is unclear whether the task results pertain specifically to caregivers or might also be similar in non-caregivers confronted with stimuli related to a loved one’s illness. Future studies using images of healthy individuals known to the loved ones might also help to disentangle whether precuneus activation in the picture factor is due to grief, familiarity or their combination.

Our confidence in the findings is bolstered by the use of a conservative statistical approach and the commonality of these results with prior neuroimaging studies. Our study confirms that there is a strong inverse relationship between caregiver grief and mindfulness, and this suggests that therapies that increase mindfulness might be specifically efficacious for helping family caregivers process the serial and compound losses that result in predeath grief reactions. Our neuroimaging findings indicate that the experience of caregiver grief has demonstrable neural substrates similar to those of bereavement with predominant involvement of midline cortical structures, and also identifies unique longitudinal brain changes associated with resolution of grief that may be studied for utility as treatment targets. Further investigation with larger samples may be useful to confirm changes in activation of these regions as biomarkers for improvement and identify whether they are specific to psychological treatment. In conclusion, mindfulness and grief, at opposing poles of the experience of clinging to transitory objects and feelings, warrant further serious study of their interrelatedness and neural mechanisms.

## Ethics Statement

This study was carried out in accordance with the recommendations of the Institutional Review Board of the University of California, Los Angeles, with written informed consent from all subjects. All subjects gave written informed consent in accordance with the Declaration of Helsinki. The protocol was approved by the Institutional Review Board of the University of California, Los Angeles.

## Author Contributions

FJ, AL, HL, and MI conceptualized the project. MA, NH and SC contributed to project administration and data curation. CC, FJ, RB-Y, LRG and LM conducted the data analysis and interpretation. FJ and CC drafted the manuscript. All authors reviewed the manuscript and provided approval of the final version.

## Conflict of Interest Statement

HL reports research support from Forest Research Institute and Allergan. AL has received research support from the National Institutes of Health, Neuronetics, Department of Defense, CHDI Foundation, and NeuroSigma, Inc. He has served as a consultant to NeoSync, Inc., Ionis Pharmaceuticals, Inc., and ElMindA. He is Chief Scientific Officer of Brain Biomarker Analytics LLC (BBA). AL owns stock options in NeoSync, Inc. and has equity interest in BBA. The remaining authors declare that the research was conducted in the absence of any commercial or financial relationships that could be construed as a potential conflict of interest.

## References

[B1] Anālayo (2003). Satipatthāna: The Direct Path to Realization. Birmingham, UK: Windhorse Publications.

[B3] Baer R. A. Smith G. T. Lykins E. Button D. Krietemeyer J. Sauer S.. (2008). Construct validity of the five facet mindfulness questionnaire in meditating and nonmeditating samples. Assessment 15, 329–342. 10.1177/1073191107313003 18310597

[B4] Bandini J. (2015). The medicalization of bereavement: (ab)normal grief in the DSM-5. Death Stud. 39, 347–352. 10.1080/07481187.2014.951498 25906168

[B5] Belden A. C. Luby J. L. Pagliaccio D. Barch D. M. (2014). Neural activation associated with the cognitive emotion regulation of sadness in healthy children. Dev. Cogn. Neurosci. 9, 136–147. 10.1016/j.dcn.2014.02.003 24646887 PMC4061244

[B6] Blandin K. Pepin R. (2017). Dementia grief: a theoretical model of a unique grief experience. Dementia 16, 67–78. 10.1177/1471301215581081 25883036 PMC4853283

[B7] Blazer D. G. (2003). Depression in late life: review and commentary. J. Gerontol. A Biol. Sci. Med. Sci. 58, M249–M265. 10.1093/gerona/58.3.M249 12634292

[B8] Bowlby J. (1973). Attachment and Loss: Volume 2. Separation, Anxiety and Anger. New York, NY: Basic Books Available online at: http://www.abebe.org.br/wp-content/uploads/John-Bowlby-Separation-Anxiety-And-Anger-Attachment-and-Loss-Vol-2-1976.pdf. Accessed January 9, 2018.

[B9] Brehmer Y. Rieckmann A. Bellander M. Westerberg H. Fischer H. Bäckman L. (2011). Neural correlates of training-related working-memory gains in old age. Neuroimage 58, 1110–1120. 10.1016/j.neuroimage.2011.06.079 21757013

[B10] Brown K. W. Coogle C. L. Wegelin J. (2016). A pilot randomized controlled trial of mindfulness-based stress reduction for caregivers of family members with dementia. Aging Ment. Health 20, 1157–1166. 10.1080/13607863.2015.1065790 26211415 PMC5070659

[B11] Chan D. Livingston G. Jones L. Sampson E. L. (2013). Grief reactions in dementia carers: a systematic review. Int. J. Geriatr. Psychiatry 28, 1–17. 10.1002/gps.3795 22407743

[B12] Chan W. C. H. Wong B. Kwok T. Ho F. (2017). Assessing grief of family caregivers of people with dementia: validation of the chinese version of the marwit-meuser caregiver grief inventory. Heal. Soc. Work 42, 151–158. 10.1093/hsw/hlx022 28575234

[B14] Collins R. N. Kishita N. (2018). The effectiveness of mindfulness- and acceptance-based interventions for informal caregivers of people with dementia: a meta-analysis. Gerontologist [Epub ahead of print]. 10.1093/geront/gny024 29635303

[B13] Collins C. Liken M. King S. Kokinakis C. (1993). Loss and grief among family caregivers of relatives with dementia. Qual. Health Res. 3, 236–253. 10.1177/104973239300300206

[B15] Corbetta M. Akbudak E. Conturo T. E. Snyder A. Z. Ollinger J. M. Drury H. A.. (1998). A common network of functional areas for attention and eye movements. Neuron 21, 761–773. 10.1016/s0896-6273(00)80593-0 9808463

[B16] Cox R. W. (1996). AFNI: software for analysis and visualization of functional magnetic resonance neuroimages. Comput. Biomed. Res. 29, 162–173. 10.1006/cbmr.1996.0014 8812068

[B17] Cox R. W. Chen G. Glen D. R. Reynolds R. C. Taylor P. A. (2017). fMRI clustering in AFNI: false-positive rates redux. Brain Connect. 7, 152–171. 10.1089/brain.2016.0475 28398812 PMC5399747

[B18] Denckla C. A. Mancini A. D. Bornstein R. F. Bonanno G. A. (2011). Adaptive and maladaptive dependency in bereavement: distinguishing prolonged and resolved grief trajectories. Pers. Individ. Dif. 51, 1012–1017. 10.1016/j.paid.2011.08.014 21984858 PMC3188454

[B19] Dickenson J. Berkman E. T. Arch J. Lieberman M. D. (2013). Neural correlates of focused attention during a brief mindfulness induction. Soc. Cogn. Affect. Neurosci. 8, 40–47. 10.1093/scan/nss030 22383804 PMC3541487

[B20] Eisenberger N. I. Lieberman M. D. Williams K. D. (2003). Does rejection hurt? An fMRI study of social exclusion. Science 302, 290–292. 10.1126/science.1089134 14551436

[B21] Engen H. G. Singer T. (2015). Compassion-based emotion regulation up-regulates experienced positive affect and associated neural networks. Soc. Cogn. Affect. Neurosci. 10, 1291–1301. 10.1093/scan/nsv008 25698699 PMC4560943

[B22] Franco C. del Mar Sola M. Justo E. (2010). Reducing psychological discomfort and overload in Alzheimer’s family caregivers through a mindfulness meditation program. Rev. Esp. Geriatr. Gerontol. 45, 252–258. 10.1016/j.regg.2010.03.006 20541288

[B23] Givens J. L. Prigerson H. G. Kiely D. K. Shaffer M. L. Mitchell S. L. (2011). Grief among family members of nursing home residents with advanced dementia. Am. J. Geriatr. Psychiatry 19, 543–550. 10.1097/JGP.0b013e31820dcbe0 21606897 PMC3101368

[B24] Gobbini M. I. Leibenluft E. Santiago N. Haxby J. V. (2004). Social and emotional attachment in the neural representation of faces. Neuroimage 22, 1628–1635. 10.1016/j.neuroimage.2004.03.049 15275919

[B25] Goldberg S. B. Tucker R. P. Greene P. A. Davidson R. J. Wampold B. E. Kearney D. J.. (2018). Mindfulness-based interventions for psychiatric disorders: a systematic review and meta-analysis. Clin. Psychol. Rev. 59, 52–60. 10.1016/j.cpr.2017.10.011 29126747 PMC5741505

[B26] Greening S. G. Osuch E. A. Williamson P. C. Mitchell D. G. V. (2014). The neural correlates of regulating positive and negative emotions in medication-free major depression. Soc. Cogn. Affect. Neurosci. 9, 628–637. 10.1093/scan/nst027 23482626 PMC4014100

[B27] Gündel H. O’Connor M. F. Littrell L. Fort C. Lane R. D. (2003). Functional neuroanatomy of grief: an fMRI study. Am. J. Psychiatry 160, 1946–1953. 10.1176/appi.ajp.160.11.1946 14594740

[B28] Hoche F. Guell X. Vangel M. G. Sherman J. C. Schmahmann J. D. (2018). The cerebellar cognitive affective/Schmahmann syndrome scale. Brain 141, 248–270. 10.1093/brain/awx317 29206893 PMC5837248

[B29] Hofmann S. G. Sawyer A. T. Witt A. A. Oh D. (2010). The effect of mindfulness-based therapy on anxiety and depression: a meta-analytic review. J. Consult. Clin. Psychol. 78, 169–183. 10.1037/a0018555 20350028 PMC2848393

[B30] Holley C. K. Mast B. T. (2009). The impact of anticipatory grief on caregiver burden in dementia caregivers. Gerontologist 49, 388–396. 10.1093/geront/gnp061 19386826

[B31] Huang F.-Y. Hsu A.-L. Hsu L.-M. Tsai J.-S. Huang C.-M. Chao Y.-P.. (2019). Mindfulness improves emotion regulation and executive control on bereaved individuals: an fMRI study. Front. Hum. Neurosci. 12:541. 10.3389/fnhum.2018.00541 30745865 PMC6360180

[B32] Huber P. J. (1964). Robust estimation of a location parameter. Ann. Math. Stat. 35, 73–101. 10.1214/aoms/1177703732

[B33] Jain F. A. Fonagy P. (2018). Mentalizing imagery therapy: theory and case series of imagery and mindfulness techniques to understand self and others. Mindfulness 1–13. 10.1007/s12671-018-0969-1 PMC7009316 32042350

[B34] Jain F. A. Nazarian N. Lavretsky H. (2014). Feasibility of central meditation and imagery therapy for dementia caregivers. Int. J. Geriatr. Psychiatry 29, 870–876. 10.1002/gps.4076 24477920 PMC4106977

[B35] Jain F. A. Walsh R. N. Eisendrath S. J. Christensen S. Rael Cahn B. (2015). Critical analysis of the efficacy of meditation therapies for acute and subacute phase treatment of depressive disorders: a systematic review. Psychosomatics 56, 140–152. 10.1016/j.psym.2014.10.007 25591492 PMC4383597

[B36] Kabat-Zinn J. (1994). Wherever You Go, There You Are: Mindfulness Meditation in Everyday Life. 1st Edn. New York, NY: Hyperion.

[B37] Kim H. (2012). A dual-subsystem model of the brain’s default network: self-referential processing, memory retrieval processes and autobiographical memory retrieval. Neuroimage 61, 966–977. 10.1016/j.neuroimage.2012.03.025 22446489

[B38] Kroenke K. Spitzer R. L. Williams J. B. W. (2001). The PHQ-9: validity of a brief depression severity measure. J. Gen. Intern. Med. 16, 606–613. 10.1046/j.1525-1497.2001.016009606.x 11556941 PMC1495268

[B39] Kübler-Ross E. (1969). On Death and Dying. New York, NY: Scribner.

[B40] Kuyken W. Warren F. C. Taylor R. S. Whalley B. Crane C. Bondolfi G.. (2016). Efficacy of mindfulness-based cognitive therapy in prevention of depressive relapse. JAMA Psychiatry 73, 565–574. 10.1001/jamapsychiatry.2016.0076 27119968 PMC6640038

[B41] Lieberman M. D. Eisenberger N. I. (2015). The dorsal anterior cingulate cortex is selective for pain: results from large-scale reverse inference. Proc. Natl. Acad. Sci. U S A 112, 15250–15255. 10.1073/pnas.1515083112 26582792 PMC4679028

[B42] Liew T. M. Yeap B. I. Koh G. C. H. Gandhi M. Tan K. S. Luo N.. (2018). Detecting predeath grief in family caregivers of persons with dementia: validity and utility of the marwit-meuser caregiver grief inventory in a multiethnic asian population. Gerontologist 58, e150–e159. 10.1093/geront/gnx097 28633382 PMC5946851

[B43] Lindauer A. Harvath T. A. (2014). Pre-death grief in the context of dementia caregiving: a concept analysis. J. Adv. Nurs. 70, 2196–2207. 10.1111/jan.12411 24702153

[B44] Liu Z. Chen Q.-L. Sun Y.-Y. (2017). Mindfulness training for psychological stress in family caregivers of persons with dementia: a systematic review and meta-analysis of randomized controlled trials. Clin. Interv. Aging 12, 1521–1529. 10.2147/CIA.s146213 29026290 PMC5626236

[B45] Mancini A. D. Sinan B. Bonanno G. A. (2015). Predictors of prolonged grief, resilience and recovery among bereaved spouses. J. Clin. Psychol. 71, 1245–1258. 10.1002/jclp.22224 26394308

[B46] Marwit S. J. Meuser T. M. (2005). Development of a short form inventory to assess grief in caregivers of dementia patients. Death Stud. 29, 191–205. 10.1080/07481180590916335 15816111

[B47] Meuser T. M. Marwit S. J. (2001). A comprehensive, stage-sensitive model of grief in dementia caregiving. Gerontologist 41, 658–670. 10.1093/geront/41.5.658 11574711

[B48] Mier D. Bailer J. Ofer J. Kerstner T. Zamoscik V. Rist F.. (2017). Neural correlates of an attentional bias to health-threatening stimuli in individuals with pathological health anxiety. J. Psychiatry Neurosci. 42, 200–209. 10.1503/jpn.160081 28234209 PMC5403665

[B49] Neimeyer R. A. Klass D. Dennis M. R. (2014). “Mourning, meaning and memory: individual, communal and cultural narration of grief,” in Meaning in Positive and Existential Psychology, eds Batthyany A. Russo-Netzer P. (New York, NY: Springer New York), 325–346.

[B50] Norouzi M. Golzari M. Sohrabi F. (2014). Effectiveness of mindfulness based cognitive therapy on the quality of life, depression and burden of demented women caregivers. Zahedan J. Res. Med. Sci. 16, 5–11.

[B51] O’Connor M. F. Wellisch D. K. Stanton A. L. Eisenberger N. I. Irwin M. R. Lieberman M. D. (2008). Craving love? Enduring grief activates brain’s reward center. Neuroimage 42, 969–972. 10.1016/j.neuroimage.2008.04.256 18559294 PMC2553561

[B52] Oken B. S. Fonareva I. Haas M. Wahbeh H. Lane J. B. Zajdel D.. (2010). Pilot controlled trial of mindfulness meditation and education for dementia caregivers. J. Altern. Complement. Med. 16, 1031–1038. 10.1089/acm.2009.0733 20929380 PMC3110802

[B53] Ong A. D. Bergeman C. S. Bisconti T. L. Wallace K. A. (2006). Psychological resilience, positive emotions and successful adaptation to stress in later life. J. Pers. Soc. Psychol. 91, 730–749. 10.1037/0022-3514.91.4.730 17014296

[B54] Pearlin L. I. Mullan J. T. Semple S. J. Skaff M. M. (1990). Caregiving and the stress process: an overview of concepts and their measures. Gerontologist 30, 583–594. 10.1093/geront/30.5.583 2276631

[B55] Postle B. R. Awh E. Jonides J. Smith E. E. D’Esposito M. (2004). The where and how of attention-based rehearsal in spatial working memory. Cogn. Brain Res. 20, 194–205. 10.1016/j.cogbrainres.2004.02.008 15183391

[B56] Prigerson H. G. Maciejewski P. K. Reynolds C. F. Bierhals A. J. Newsom J. T. Fasiczka A.. (1995). Inventory of complicated grief: a scale to measure maladaptive symptoms of loss. Psychiatry Res. 59, 65–79. 10.1016/0165-1781(95)02757-2 8771222

[B57] R Core Team (2016). R Core Team R. Vienna: R Foundation for Statistical Computing Available online at: http://www.r-project.org. Accessed October 31, 2016.

[B58] Ramon M. Gobbini M. I. (2018). Familiarity matters: a review on prioritized processing of personally familiar faces. Vis. Cogn. 26, 179–195. 10.1080/13506285.2017.1405134

[B59] Rando T. A. Doka K. J. Fleming S. Franco M. H. Lobb E. A. Parkes C. M.. (2012). A call to the field: complicated grief in the DSM-5. Omega 65, 251–255. 10.2190/OM.65.4.a 23115891

[B60] Rush A. J. Trivedi M. H. Ibrahim H. M. Carmody T. J. Arnow B. Klein D. N.. (2003). The 16-item quick inventory of depressive symptomatology (QIDS), clinician rating (QIDS-C) and self-report (QIDS-SR): a psychometric evaluation in patients with chronic major depression. Biol. Psychiatry 54, 573–583. 10.1016/s0006-3223(02)01866-8 12946886

[B61] Saad Z. S. Glen D. R. Chen G. Beauchamp M. S. Desai R. Cox R. W. (2009). A new method for improving functional-to-structural MRI alignment using local pearson correlation. Neuroimage 44, 839–848. 10.1016/j.neuroimage.2008.09.037 18976717 PMC2649831

[B62] Schilbach L. Wohlschlaeger A. M. Kraemer N. C. Newen A. Shah N. J. Fink G. R.. (2006). Being with virtual others: neural correlates of social interaction. Neuropsychologia 44, 718–730. 10.1016/j.neuropsychologia.2005.07.017 16171833

[B63] Sheehan D. V. Lecrubier Y. Sheehan K. H. Amorim P. Janavs J. Weiller E.. (1998). The mini-international neuropsychiatric interview (M.I.N.I.): the development and validation of a structured diagnostic psychiatric interview for DSM-IV and ICD-10. J. Clin. Psychiatry 59, 34–57. 9881538

[B64] Shuter P. Beattie E. Edwards H. (2014). An exploratory study of grief and health-related quality of life for caregivers of people with dementia. Am. J. Alzheimers Dis. Other Demen. 29, 379–385. 10.1177/1533317513517034 24381138 PMC10852965

[B65] Sikder A. Yang F. Schafer R. Dowling G. A. Traeger L. Jain F. A. (2019). Mentalizing imagery therapy mobile app to enhance the mood of family dementia caregivers: feasibility and limited efficacy testing. JMIR Aging 2:e12850 10.2196/preprints.12850 PMC6715046 31518275

[B66] Sokolov A. A. Miall R. C. Ivry R. B. (2017). The cerebellum: adaptive prediction for movement and cognition. Trends Cogn. Sci. 21, 313–332. 10.1016/j.tics.2017.02.005 28385461 PMC5477675

[B67] Van Dam N. T. van Vugt M. K. Vago D. R. Schmalzl L. Saron C. D. Olendzki A.. (2018). Mind the hype: a critical evaluation and prescriptive agenda for research on mindfulness and meditation. Perspect. Psychol. Sci. 13, 36–61. 10.1177/1745691617709589 29016274 PMC5758421

[B68] Venables W. N. Ripley B. D. (2002). Modern Applied Statistics with S. 4th Edn. New York, NY: Springer.

[B69] Völlm B. A. Taylor A. N. W. Richardson P. Corcoran R. Stirling J. McKie S.. (2006). Neuronal correlates of theory of mind and empathy: a functional magnetic resonance imaging study in a nonverbal task. Neuroimage 29, 90–98. 10.1016/j.neuroimage.2005.07.022 16122944

[B70] Wager T. D. Keller M. C. Lacey S. C. Jonides J. (2005). Increased sensitivity in neuroimaging analyses using robust regression. Neuroimage 26, 99–113. 10.1016/j.neuroimage.2005.01.011 15862210

[B71] Walsh F. McGoldrick M. (1998). “A family systems perspective on loss, recovery and resilience,” in Working With the Dying and Bereaved, eds Utcliffe P. Tufnell G. Cornish U. (London: Macmillan Education UK), 1–26.

[B72] Whitebird R. R. Kreitzer M. Crain A. L. Lewis B. A. Hanson L. R. Enstad C. J. (2013). Mindfulness-based stress reduction for family caregivers: a randomized controlled trial. Gerontologist 53, 676–686. 10.1093/geront/gns126 23070934 PMC3709844

[B73] Witthöft M. Mier D. Ofer J. Müller T. Rist F. Kirsch P.. (2013). Neuronal and behavioral correlates of health anxiety: results of an illness-related emotional stroop task. Neuropsychobiology 67, 93–102. 10.1159/000345545 23296017

